# Man and His Enemies

**Published:** 1889-03-16

**Authors:** 


### THE DOCTOR'S PULPIT.

### No. II.—MAN AND HIS ENEMIES.

### INHERITED TAINTS.

It seems hard that a human creature should come into the world bringing with him injuries inflicted by unwitting enemies before he was born. It is tragic that those enemies should not seldom be his own parents. Yet such is the case. Many children are born who do not survive more than a few days or weeks, solely because they have inherited a specific taint, which has been in them so potent as to kill. The present writer could quote a number of examples of this from his own experience, and every observant medical man of mature age could confirm it abundantly. Even when infant life is not destroyed, certain surviving children are endowed with unwholesome and injured constitutions, which enfeeble their vital powers, make them less able to resist disease when it comes, and not seldom are the source and origin of obscure maladies which may threaten or even finally end in death.

It is the fashion among many persons to pass lightly over topics of this kind, or to exclude them altogether from discussion. That is criminal, and stupid too. If we were sure ours would be the last generation of men, or if science had reached her ultimatum of practical knowledge and wisdom, so that no improvement in the world's condition could reasonably be looked for in the future, then might we say with the feeble pessimist that "things are too bad to be mended, and that, therefore, discussion is useless." But pessimism is the creed of ricketty intellects, nerveless wills, and craven hearts. The true state of the matter is, that we are but at the beginning of the age of science, and that science, if intelligently pursued and rationally used, will bring about a condition of things as much superior to the present as the present is to the period of the cave man.

In the world's history different epochs have been marked by upheavals which have indicated the commencement of new developmental departures. The decline and fall of the Roman Empire, under the resistless force of the Christian revolution, was the outward and visible evidence of the waking of new moral and spiritual energies, which were as permanent as they were omnipotent. They were permanent because they had become part of the constitution of developing man. Another stage of human advancement was reached when in the fourteenth and fifteenth centuries whole peoples claimed for themselves the unlimited freedom of individual opinion. This century has gained a still higher platform; and to-day every thinking man is demanding to know the very ground and nature of his own being and personality. Religion and philosophy have for centuries been giving their partial answers to his questionings. But now the physical man is being interrogated: his brain, his organs, his constitution, his diseases, his normal and his abnormal states—all are asked to contribute their quota of light towards the illumination of the great enigma; What is man? Whence is he? Why is he? What shall he be?

The science of physiology, of which pathology and medicine are but branches, and which, properly speaking, includes both mind and morals, is a sun which illuminates a thousand regions, which, but for its rays, must have remained for ever obscure. Unhappily, physiology has the misfortune to be associated almost exclusively with medicine. Many medical men, unable to see the great part it has to play in the world, have tried to yoke it to their own triumphal car, and to make it merely a subordinate branch of their limited art. Every physiologist is charged with the duty of preventing such a degradation, and saving for the world the true and complete "science of man." Physiology strives to explore the very inmost recesses of being. It watches the inception of the physical organism—watches and compares. It marks progress from day to day, and even from hour to hour. It notes every circumstance which can by any possibility influence the organism, or help to determine its character and development. So far as human powers may prevail, physiology steadily conquers knowledge. What are the results?

One result of the labours of physiologists has been the clearing of the mental vision, and the gradual comprehension of the great, pervasive, and potential fact of "heredity." "The sins of the fathers shall be visited upon the children," said Moses, more than three thousand years ago. Probably he comprehended in but a very small measure the significance of his own utterance. Not only do parents transmit to children their mental peculiarities, their moral tendencies, the features of the face, the stoop of the shoulders, and the trick of the gait, but they pass on to them their blood, their brain, their glands, their very soul and life. We do not mean to say that heredity is a tyrant from which there is no escape, and that as is the parent in constitution and conduct, so also must be the children to the remotest generation. If that were one of the discoveries of physiology, small thanks would be due to the science from overburdened man. But it is not so. The parent himself, as is well-known, can modify and make worse or better both his constitution and his character. Similarly, the child's constitution and character may be changed, until, by the operation of the law of heredity itself, a not very remote descendant may be the antipodes of his early progenitors. The discovery of an existing inherited taint of disease or of vice in a child is not a cause for regret, but for thankfulness. The disease taint itself is, of course, to be deplored, and so is the inherited vice; but its early discovery is to be hailed with gratitude as pointing out lines of physical and moral treatment which may lead to the practical enfeeblement of the taint, or even to its eradication.

Civilised man may be separated into distinct classes according to certain prevailing family conditions and tendencies. For example, there is the gouty class, the rheumatic class, the strumous class, the phthisical class, the specifically-tainted class already mentioned, and others. Some persons are born, not merely with tendencies to gout, struma, and so on, but with the whole of their organs and tissues already in a limited measure gouty and strumous. Many a gouty person may pass the whole of life without having a single attack of acute gout, and yet the gout shall be a part of the constitution, and shall influence health, spirits, and working power in a very marked degree. The recognition of this fact by the physician, as Trousseau well shows, is of the first importance in medical practice; but it is equally important, and perhaps even more so, that the gouty person should himself know it. For what would a really intelligent man do who knew that he was of gouty descent, and had in him seeds which, under favouring conditions, might blossom into grave and dangerous maladies? What would a man do if he knew he were descended from an ancestry of liars? If he wanted to be a true man, he would resolve not only to tell no lie himself so long as life endured, but to leave no stone unturned until the vicious habit was eradicated, both from himself and any children he might have. Similarly, if a man born gouty be really a man of intelligence and conscience, he will take care to spend his life in such a way as that, if possible, the gouty taint shall be eradicated from his own constitution, and if not completely subdued, that it shall at least be diminished in potency, and that the next generation shall be much more wholesome and vigorous than himself.

Intellectual babies and medical men of vulgar minds may consider this a "counsel of impossible perfection." But if science means anything practical at all for the world, it means that the rational counsels of perfection of to-day are to be the commonplaces of daily practice to-morrow. There is such a thing as scientific infidelity, which is as withering and deadly in its influence as every other kind of infidelity. Science, it cannot be too often repeated—science, and *all* science, is for the world, not for the chosen few. Any science that cannot be communicated to the world is self-reprobated and worthless. The man of true scientific temper can never be content until he has shared his knowledge with another, and until the other shares his own enthusiasm. Selfishness in scientific knowledge is like every other kind of selfishness: it comes from the devil, and should be hounded out of the world by every honest and wholesome-hearted man. The facts of heredity in their widest bearing reveal many enemies of man dating from his birth, enemies which are with difficulty destroyed by the most strenuous efforts of a long and wisely-spent life. It is alike the interest and the duty of every thoughtful man to make himself acquainted with these facts, and to live his life on such lines as plain sense and honest intent would dictate.

